# Supplementary material for: Right Ventricle and Epigenetics: A Systematic Review
Source: Cells. 2023 Nov 23;12(23):2693. doi: 10.3390/cells12232693 (PMC10705252; doi:10.3390/cells12232693)
Supplement: Supplementary file 1 [file cells-12-02693-s001.zip › cells-2657671-supplementary.pdf]

Figure S1

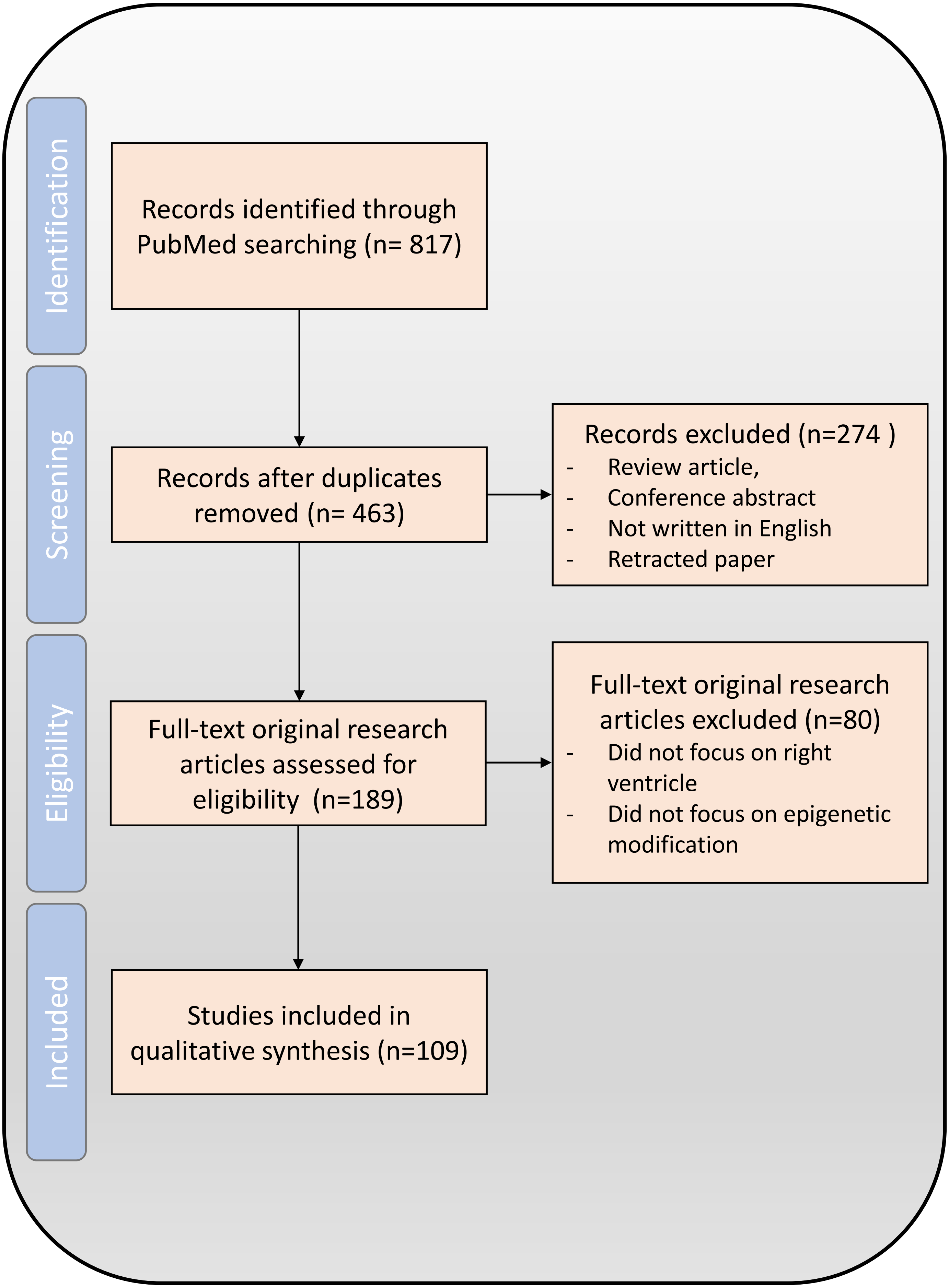

Identification
Screening
Eligibility
Included
Records identified through PubMed searching (n= 817)
Records after duplicates removed (n= 463)
Records excluded (n=274)
- Review article,
- Conference abstract
- Not written in English
- Retracted paper
Full-text original research articles assessed for eligibility (n=189)
Full-text original research articles excluded (n=80)
- Did not focus on right ventricle
- Did not focus on epigenetic modification
Studies included in qualitative synthesis (n=109)

Table S1

Group	Article	Review	Original	Excluded	Included
epigenet* AND right ventri*	90	30	60	62	28
right ventri* AND histon*	67	11	56	29	38
right ventri* AND embryo* AND epigenet*	20	4	16	11	9
right ventri* AND DNA methyl*	36	7	29	23	13
right ventri* AND methyl* AND DNA*	58	9	49	35	23
right ventri* AND long non coding RNA	33	8	25	18	15
epigenet* AND right ventri* AND LncRNA*	2	1	1	1	1
right ventri* AND LncRNA*	22	5	17	14	8
epigenet* and right ventri* AND therap*	40	23	17	28	12
right ventri* AND chromati*	65	11	54	41	24
right ventri* AND long non coding	16	5	11	9	7
right ventri* AND non coding*	53	13	40	38	15
right ventri* AND micro-RNA	5	4	1	3	2
right ventri* AND mir*	149	4	145	70	79
right ventri* AND microRNA*	161	19	142	84	77
**TOTAL**	817	154[#]	663[#]	466[#]	351[#]

[#] *Includes duplicates article*
